# Supplementary material for: Growth and Phenology of Vulpia Myuros in Comparison with Apera Spica-Venti, Alopecurus Myosuroides and Lolium Multiflorum in Monoculture and in Winter Wheat
Source: Plants (Basel). 2020 Nov 5;9(11):1495. doi: 10.3390/plants9111495 (PMC7694455; doi:10.3390/plants9111495)

Supplementary files

Table S1. Estimates and 95 % bootstrap confidence intervals of thermal times (°C) from sowing to various phenological stages for grasses and crop in 2017/18.

Species	BBCH 13	BBCH 21 [b]	BBCH 31	BBCH 51	BBCH 80	BBCH 93
V. myuros	356.9 (346.5-362.4) b	392.5 (381.6-397.9) a	788.8 (765.5-825.5) c	927 (896.4-966.1) c	1342.4 (1316.6-1371.4) b	1567 (1547.7-1613.8) b
A. spica-venti	355.7 (346.5-362.6) b		872 (848.5-886.5) d	1174.1 (1147.4-1216.2) e	1522.2 (1506-1544.3) d	1689.8 (1673.6-1701.8) c
A. myosuroides	334.9 (327.3-351.6) b		654 (627.1-678.1) a	758.4 (739.6-794.7) a	1209.7 (1204.1-1247.9) a	1387.5 (1371.8-1392.4) a
L. multiflorum	341.4 (329.8-353.5) b		740.9 (716.4-750.9) b	867.3 (848.5-887) b	1351.6 (1322.7-1375) b	1646 (1626.1-1681.8) c
Winter wheat	291.9 (288-295.3) a		712.2 (704.9-728.7) b	1035.7 (1029.4-1050.8) d	1382.3 (1375.6-1388.6) c	
Significance levels [a]	$P < 0.00001$	$P=0.125$	$P < 0.00001$	$P < 0.00001$	$P < 0.00001$	$P < 0.00001$

[a] Within each growth stage, species with the same letter do not differ significantly in thermal time needed to achieve a specific development stage. The mean thermal times were compared between the species by a permutation test at 5% significance level.
[b] The time for attaining the BBCH 21 stage was estimated similar for all plant species.

Table S2. Estimates and 95 % bootstrap confidence intervals of thermal times (°C) from sowing to various phenological stages for grasses and winter wheat in 2018/19.

Species	BBCH 13	BBCH 21	BBCH 31	BBCH 51	BBCH 80	BBCH 93
V. myuros	330.8 (323.5-348.8) b	361.5 (338.1-374.7) ab	1161.4 (1138.7-1169.5) c	1353.7 (1345.4-1364.8) b	1845.3 (1821.5-1872.2) b	2164.7 (2131.5-2186.7) b
A. spica-venti	357.4 (337.7-374.2) b	463.7 (447.4-474.4) c	1258.3 (1252.2-1270.9) d	1575.1 (1565.4-1594) d	2184.6 (2147.7-2195.8) d	2249.1 (2219.1-2256.8) c
A. myosuroides	339 (326.6-360.4) b	374.3 (348.1-390.4) b	881.3 (856.6-938.3) a	1125.2 (1095.3-1133.1) a	1634.3 (1616.6-1650.6) a	1869.6 (1839.7-1908.3) a
L. multiflorum	332.4 (313.4-342.9) b	385.8 (364.8-405.6) b	988.5 (930.8-1029.2) b	1368.2 (1354.4-1377.6) b	1832.9 (1818.3-1864.8) b	2213.2 (2175.3-2221.5) bc
Winter wheat	275 (266.9-284.5) a	331.6 (326.8-343.5) a	1145.8 (1135.3-1159.4) c	1434.4 (1430.3-1449.7) c	1970.4 (1957.8-1989.2) c	
Significance levels [a]	$P < 0.00001$	$P < 0.00001$	$P < 0.00001$	$P < 0.00001$	$P < 0.00001$	$P < 0.00001$

[a] Within each growth stage, species with the same letter do not differ significantly in thermal time needed to achieve a specific development stage. The mean thermal times were compared between the species by a permutation test at 5% significance level

Figure S1. Biomass production of target plants the *V. myuros* (open markers) and *A. spica-venti* (filled markers) in response to increasing densities of winter wheat when transplanted at 2-leaf stage (circles) and 3-4 leaf (squares) stages of winter wheat in 2017/18 (A and C) and 2018/19 (B and D). Data from first harvest (A and B) and second harvest (C and D) are shown with fitted curves.

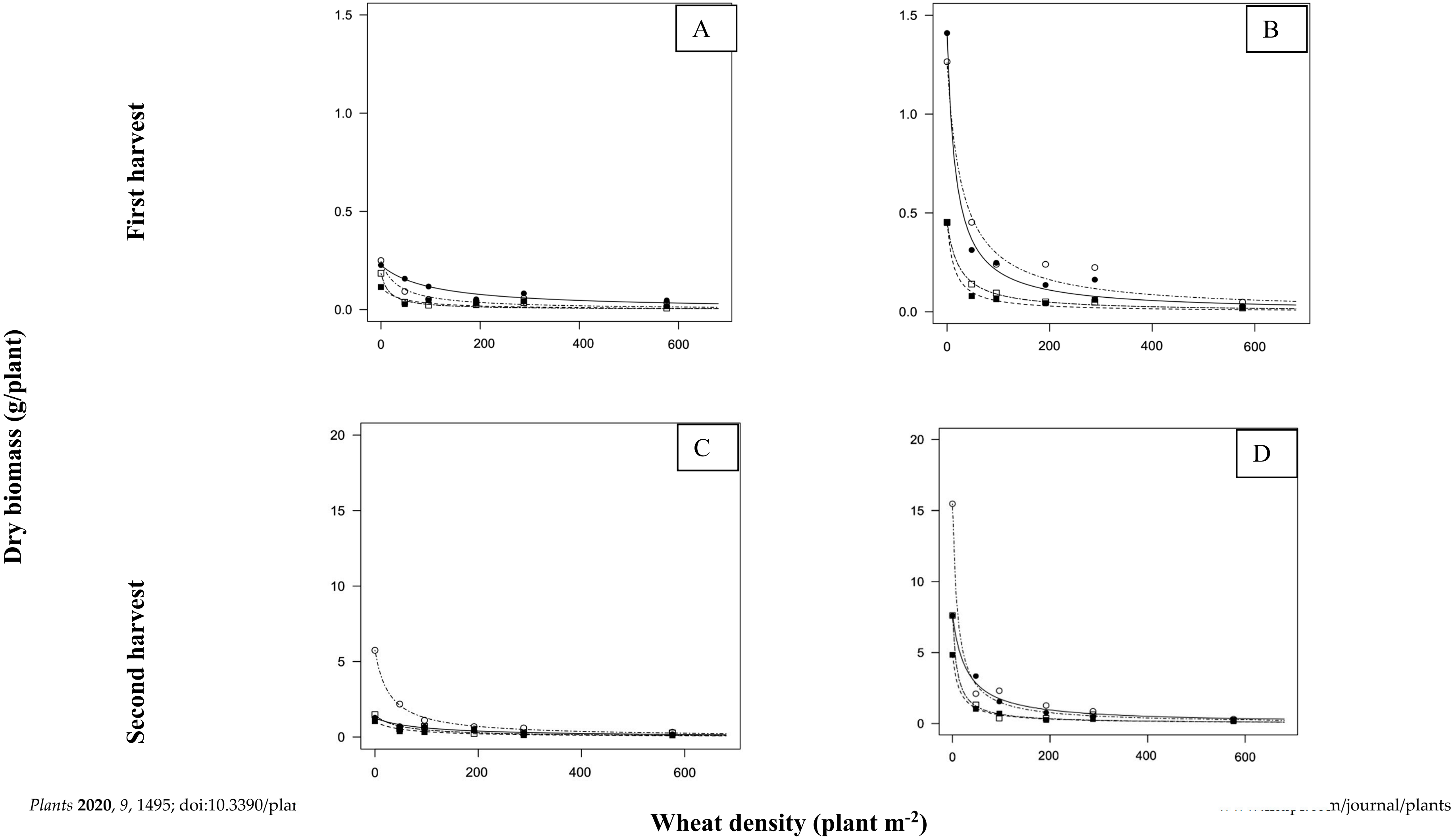

1495; doi:10.3390/plants9111495Figure S2. Response of target plants the *V. myuros* (open markers) and *A. spica-venti* (filled markers) with increasing densities of winter wheat when transplanted at 2-leaf stage (circles) and 3-4 leaf (squares) stages of winter wheat in 2017/18 (A and C) and 2018/19 (B and D). Data of biomass production (A and B) and seed production (C and D) are presented at third harvest.

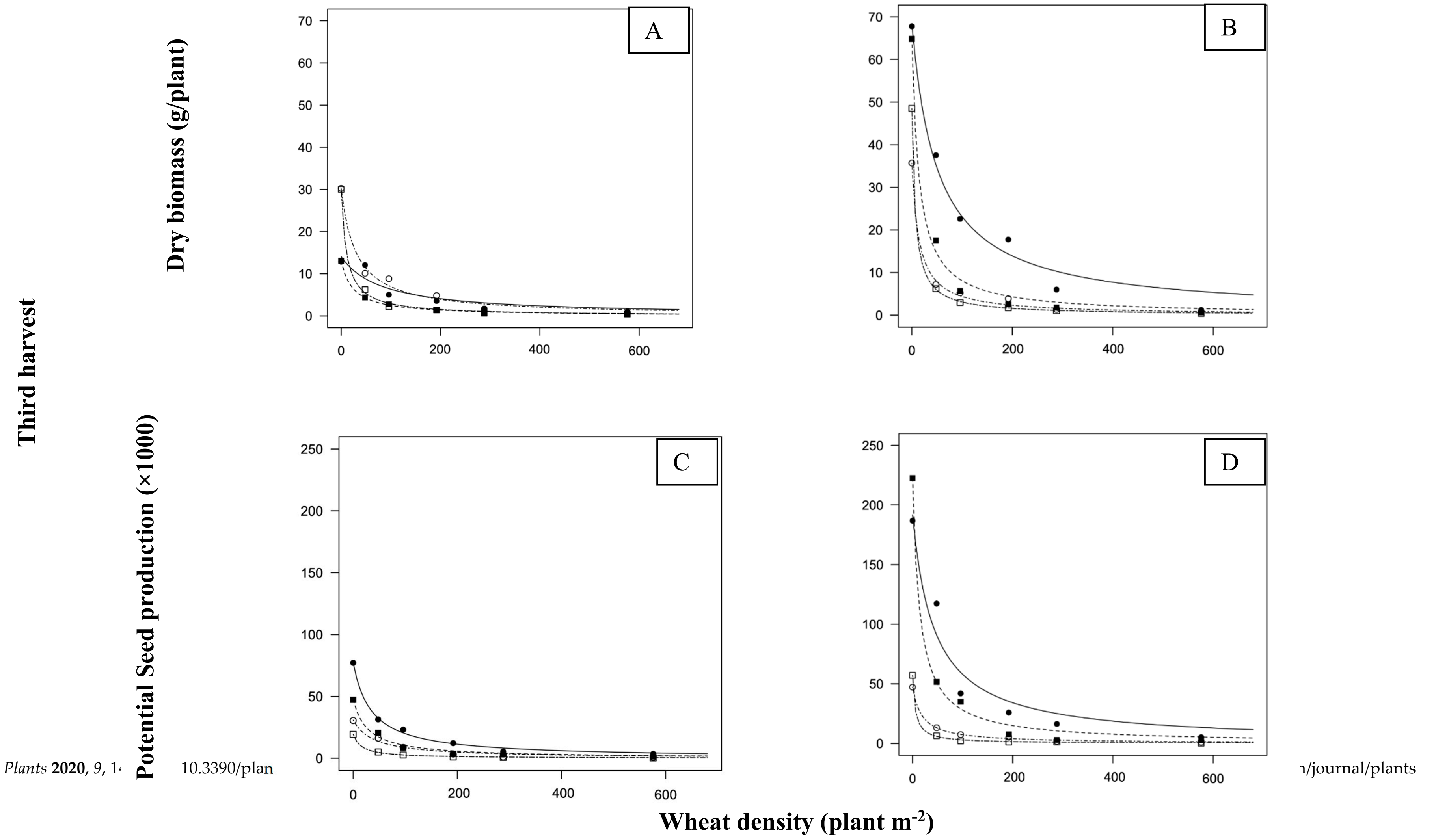

Supplement: Supplementary file 1 [file plants-09-01495-s001.pdf]
